# Longitudinal trends and predictors of limitations in activities of daily living in community-dwelling older adults: evidence from the KLoSA study

**DOI:** 10.3389/fpubh.2024.1485732

**Published:** 2024-12-13

**Authors:** Eunmi Oh, SeolHwa Moon, Gwi-Ryung Son Hong

**Affiliations:** ^1^Research Institute of Nursing Science, Hanyang University, Seoul, Republic of Korea; ^2^Department of Nursing, Hoseo University, Cheonan-si, Republic of Korea; ^3^College of Nursing, Hanyang University, Seoul, Republic of Korea

**Keywords:** activities of daily living, functional limitation, older adults, longitudinal studies, predictors, bathing, assistive technology

## Abstract

**Background:**

As life expectancy increases, the number of older adults with functional limitations is also increasing. Functional limitations are associated with adverse health outcomes such as reduced independence, diminished quality of life (QoL), and disability. Therefore, identifying which activities of daily living (ADLs) are limiting and understanding the influencing factors are crucial for developing tailored interventions. Although various factors influence ADL limitations, few studies have identified the longitudinal factors associated with each ADL. This study explores the longitudinal trends and factors associated with the ADL total score and functional limitations with each ADL among older adults in Korea.

**Methods:**

Using data from the Korean Longitudinal Study of Aging (KLoSA) from 2006 to 2020, we analyzed 1,388 people aged 65 and older who had no ADL limitations in 2006. An ADL limitation was defined as partial or complete dependence in any of the following ADLs: getting dressed, washing face and hands, bathing, eating, transferring, toileting, and continence. We used repeated measures analysis of variance and multivariate logistic regression to investigate the trends and predictors of ADL limitations over a 14-year period.

**Results:**

In 2006, the mean age of the participants was 69.88 years (SD = ±4.11), and 60.20% were female. The prevalence of total ADL limitations and limitations in each of the seven ADLs increased gradually during the 14 years of follow up. In 2020, the ADL items with the highest prevalence of limitations were bathing, getting dressed, and washing face and hands. The common significant predictors for total ADL limitations and limitation in the top three ADLs were age and cognitive function.

**Conclusion:**

ADL limitations among Korean older adults significantly increase over time, which highlights the need for integrated early intervention and continuous support for bathing limitations, including the application of integrated assistive technologies. In particular, because age and cognitive function were identified as the major predictors for limitations in both total ADLs and the top three ADLs, early assessment and appropriate intervention strategies need to consider those factors to prevent ADL limitations in older adults or to meet the immediate needs of those already experiencing ADL limitations. This approach could enhance the QoL for older adults and contribute to the development of long-term healthcare plans.

## 1 Introduction

As the population of older adults increases worldwide, the number of older adults with functional limitations is also increasing (1, 2). Functional limitations impede the older adult’s freedom in daily life and reduce their independence. Compared to older adults without functional limitations, those with functional limitations generally have more serious health problems, limited social participation, greater economic vulnerability, and less access to medical services (3). Older adults with functional limitations also tend to develop additional health problems due to aging (4, 5), which increases their physical and mental difficulties and leads to negative health outcomes such as depression (6) and decreased quality of life (QoL) (7). In addition, a study on 7-year follow-up of Dutch community-dwelling older adults aged 75 years and older, functional limitation predict mortality (8). The burden of care and high medical costs caused by the increasing number of older adults with functional limitations are affecting both individual and social resources (1). Therefore, it is increasingly important to understand the health status and needs of older people and target public health actions based on that understanding (9). The World Health Organization (WHO) emphasizes that measuring, monitoring, and understanding the health of older populations is essential to optimizing their functional capacity and adapting health systems to their needs (10).

In this context, identifying the Activities of Daily Living (ADLs) with the greatest functional limitations is crucial for developing and implementing appropriate assistive technology to support independent l living in older adults and reducing caregiving burdens. *Assistive technology* refers to a broad category of assistive products, related systems, and services. Assistive technology enables and facilitates the inclusion, engagement, and participation of individuals who are older adults, disabled, or chronically ill in all sectors of society, including family, community, political, economic, and social spheres (11). In response to the increasing number of older adults with functional limitations due to the aging of the global population, interest is growing in developing assistive technologies to improve independent living and reduce caregiving burdens for older adults with functional limitations (12, 13). For example, the effects of care robots on the ADLs among older people with functional limitations (e.g., improving ADL scores and convenience) (14) and their effects on care service stakeholders (15) are being actively studied.

In addition, research on the determinants of functional limitations in the older adults is being actively conducted to plan future healthcare services. The factors that influence functional limitations are diverse. According to a systematic review of studies of community dwelling older adults (16), the relevant sociodemographic factors are old age, being female, living alone, and having a low income or low educational state. The relevant physical health–related factors are multimorbidity, poor self-rated health, falls, polypharmacy, pain, vision impairment, hearing impairment, low grip strength, obesity, and reduced physical activity. The mental health factors are cognitive decline, depression, and reduced QoL, and the psychosocial factors are decreased social participation and social isolation.

As such, functional limitation is a complex and multifactorial condition affected by various risk factors, so it is difficult to measure accurately (1, 16). Although a variety of tools to measure functional limitation have been developed, no unified standards or methods have been established for assessing and classifying disability in older adults (16). The tool most commonly used to assess the functional limitations of older adults evaluates their ability to perform ADLs, which reflect the basic minimum self-care skills that an individual needs for independent living, including dressing, eating, and toileting. When a person’s ability to perform ADLs is impaired, they can experience serious difficulties in surviving daily life independently, and they can have poor QoL (7). Therefore, assessing the ability of older adults to perform ADLs plays an important role in identifying their health needs and vulnerabilities (9, 10).

However, most previous studies classified people with functional limitations into groups according to their total scores on ADL tests (17, 18) or into dichotomous groups based on the presence or absence of functional limitations (19, 20). Even in studies that analyzed each ADL, the frequency or change pattern for each item was given only according to gender and age (21), or the data were cross-sectional (7, 17, 20, 22). Longitudinal studies on the factors that influence the ability to perform each ADL are rare. It is important to identify changes in the ability to perform each ADL and the factors that influence those changes because we need to understand which ADLs are most likely to become difficult as they age and require care. To provide direction for care-related environments and policies, results from total ADL scores have limited value. By examining the degree to which older adult people have functional limitations in each ADL, we can enable care-related policies to be tailored to the ADLs with the most severe functional limitations, and the care industry can prioritize its product development.

If the functional limitations of older adults persist or become severe, they can lead to disability (23, 24). Therefore, longitudinal research is needed to predict and prepare for future healthcare needs by understanding how social, economic, and demographic changes affect the health and disability of older adults with functional limitations over time. For example, to develop care robots or devices suitable for helping older adults with functional ADL limitations and thereby reduce the physical burden of caregivers, the specific needs of those older adults must be recognized (13). This previous study (13), using a mixed-methods approach and a public-private cooperation model, identified 9 types of nursing robots and 5 key research and development strategies suitable for the nursing field in Korea through user participation research. It provided a foundation for understanding the needs and priorities of older adults with functional limitations and their caregivers (13). In other words, the development of complex systems (e.g., smart home, mobile robot platforms with daily activity recognition) requires research into the specificities of daily life limitations (14). In the process of determining the type of care robots that older adults with functional limitations need and then developing and introducing those robots, detailed longitudinal information about each ADL is needed more than information about total ADL scores.

As Korea’s population is aging fastest among Organization for Economic Cooperation and Development countries (25), the proportion of the old adults with disabilities is increasing. According to the National Survey on Persons with Disabilities, conducted every 3 years in South Korea, the proportion of older adults with disabilities aged 65 years and older increased from 49.9% in the 2020 survey to 54.3% in 2023 (26). Korea is experiencing complex challenges in the fields of healthcare, family support, employment, and community participation due to the increase in the number of old adults with a disability. A need to improve their QoL (human rights, finances, care burden, poor quality of care services, etc.) has emerged due to social problems arising from their institutionalization. Along with deinstitutionalization, older adults with a disability need assistance in daily life to maintain their independence and live in the community, and enabling those changes is an important policy task (13).

Therefore, Korea provides housing, healthcare, and nursing care so that people with functional limitations can receive services tailored to their individual needs in the place where they live and in harmony with the local community. Through the “community care policy,” efforts are being made to ensure that older adult people with functional limitations or disability are respected for their value as human beings, including the provision of inclusive care to improve their QoL (27). In addition, the Korean government selected 9 priority technology areas for care and rehabilitation to assist older adults with functional limitations with their ADLs (e.g., mobility, bathing, eating). Furthermore, Korea is making efforts at the national level to conduct research, develop service models, and build an integrated system (e.g., technology development, education, training, commercialization, platforms) to apply care and rehabilitation robot technology to care services (13, 28).

Therefore, the purpose of this study is to analyze the Korean Longitudinal Study of Aging (KLoSA) database to identify trends in ADL limitations and identify factors that affect not only the total ADL score, but also the three ADLs with the greatest limitation effects in older adults. Unlike most previous studies that focus primarily on the total ADL score, this study analyzes both the total ADL score and each individual ADL item to identify which specific functions show more pronounced limitations and the factors influencing each. This approach provides detailed and practical insights, and this study is the first in Korea to identify specific ADLs for which older adults need the most assistance, providing foundational data for the development and application of customized assistive technologies (e.g., prioritizing the development of care robots and older adult care support). This approach is distinguished from previous studies by offering a more detailed and practical analysis of ADL limitations, enabling the development of solutions that reduce caregiving burdens and support independent living for older adults.

## 2 Materials and methods

### 2.1 Sample

**Figure 1:**
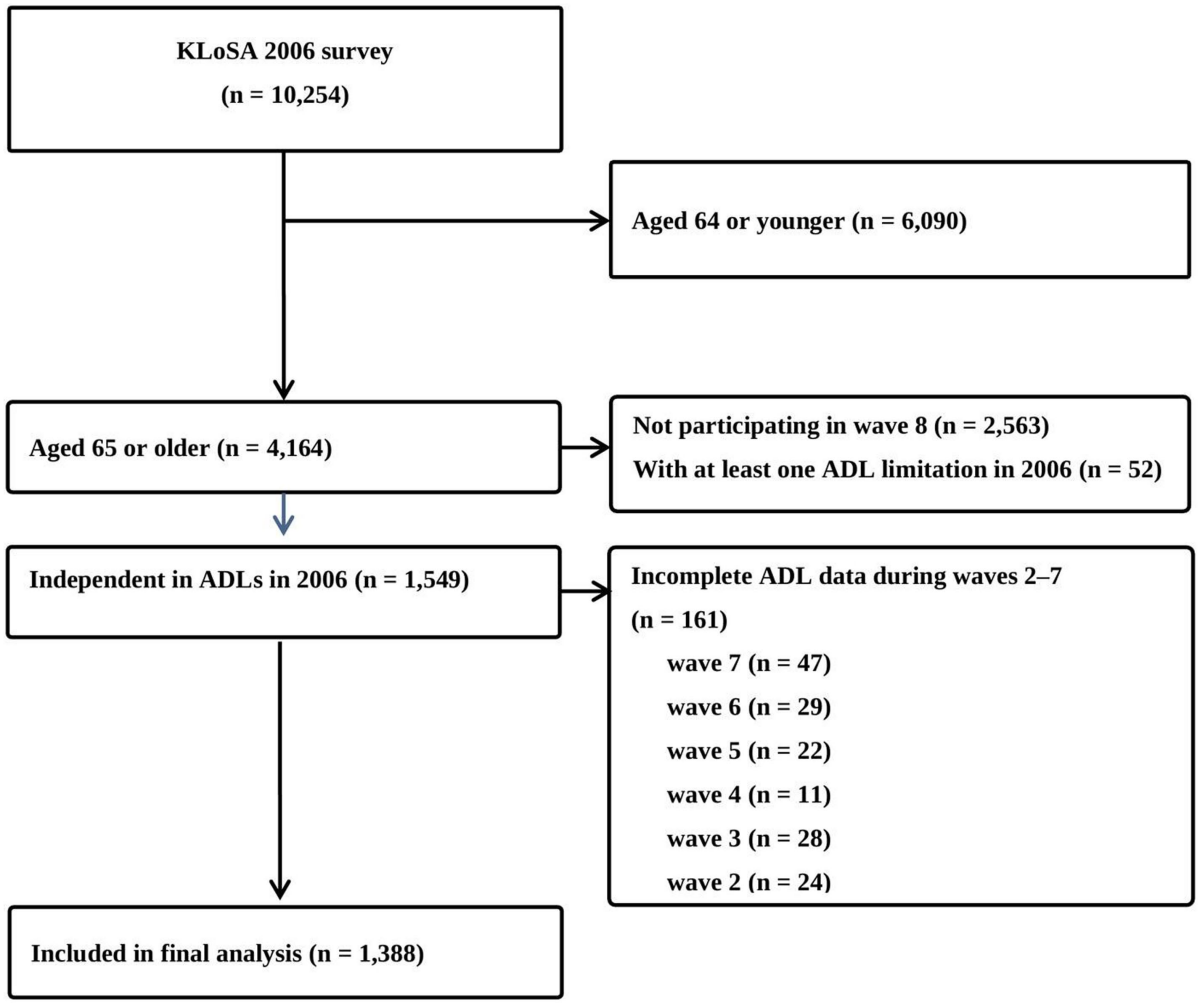
Flow chart of the study.

This secondary data analysis study was conducted using data from the ongoing panel survey, KLoSA (29). KLoSA uses a multi-stage stratified probability sampling approach to survey households across the country. The initial response rate was high at over 80% and has remained stable since then. Data is collected through face-to-face interviews based on a standardized questionnaire by trained surveyors, and the collected data undergoes multiple levels of validation to ensure the reliability and quality of the data. KLoSA has been conducted every 2 years since 2006 and data were collected from a random sample of 10,254 community-dwelling adults aged 45 and older at baseline (2006). This study used data from 8 waves (2006, 2008, 2010, 2012, 2014, 2016, 2018, and 2020) over 14 years to examine changes in ADL limitations and their associated factors in older adults. The inclusion criteria for this study were as follows: (1) aged 65 years or older at wave 1 (2006), (2) full independence in ADL items at wave 1, and (3) complete data for all eight waves. The number of participants in the final analysis was 1,388 after excluding 8,866 from the baseline (*n* = 10,254) due to (1) age younger than 65 years in 2006 (*n* = 6,090), (2) not participating in all 8 waves (*n* = 2,563), (3) at least one ADL limitation in 2006 (*n* = 52), or (4) incomplete ADL data in waves 2–7 waves (*n* = 161). A detailed study flow chart is provided in Figure 1.

### 2.2 Ethical approval and consent to participate

The KLoSA survey has been approved by Statistics Korea (approval number: 336002) and is conducted in accordance with the principles of the Declaration of Helsinki, including the collection of written informed consent from the participants. Because all data are de-identified and made publicly available for scientific research, ethical approval was not needed for this study.

### 2.3 Measurements

#### 2.3.1 Dependent variable: functional limitations

Functional limitations were assessed using the seven ADLs: getting dressing, washing face and hands, bathing, eating, transferring, toileting, and continence (30). The scores for each item dichotomize respondents as independent (score = 0) or partially or fully dependent (score = 1) in that activity. Total scores thus range from 0 to 7. In this study, an ADL limitation was defined as a total ADL score of 1 or more (31).

#### 2.3.2 Independent variables

The independent variables were demographic factors and health-related factors identified as influencing ADLs in the previous literature (16, 32). The demographic variables were age, sex, living arrangement, marital status, education, and participation in social groups. Living arrangements were classified into living “living alone,” “living with spouse,” or “living with spouse and others.” Marital status was assessed by asking, “What is your current marital status?” with response options of “Married,” “Separated/Divorced/Widowed,” or “never married.” The responses were classified into a binary variable: “Married” and “Separated/Divorced/Widowed or never married.” Participation in social groups was categorized according to involvement in any social groups (e.g., religious activities, volunteer work, social clubs, etc.). If participants indicated involvement in at least one social activity, they were classified as “yes”; otherwise, they were classified as “no.”

The health-related factors were regular exercise, number of chronic diseases, cognition, perceived hearing status, perceived vision status, perceived health, perceived QoL, fall experience, trouble with a fear of falling (FOF), grip strength, and body mass index (BMI). Regular exercise was categorized as “yes” if participants responded that they engaged in regular exercise at least once a week, and “no” otherwise. The number of chronic diseases was calculated based on hypertension, diabetes mellitus, cancer, pulmonary disease, liver disease, heart disease, cerebrovascular disease, psychiatric disease, and arthritis, all as diagnosed by a doctor. Cognition was measured using the Korean version of the Mini-Mental State Examination (K-MMSE) (33), which consists of 19 items. The total score ranges from 0 to 30, with lower scores indicating poorer cognitive function. In this study, the level of cognitive function was classified based on the total K-MMSE score as follows: dementia (0–17), mild cognitive impairment (18–23), and normal (≥ 24) (33, 34). Perceived hearing (with hearing aids, if used) and vision (including corrected vision) in daily life was reclassified based on participant responses: “very good” and “good” were categorized as “good,” and “fair,” “poor,” and “very poor” were categorized as “poor.” Perceived health was similarly reclassified: “very good” and “good” were categorized as “good,” and “fair,” “poor,” and “very poor” were categorized as “poor.” Perceived QoL was assessed by asking participants to rate their overall life satisfaction on a scale from 0 to 100 (the closer to 100, the higher the perceived QoL), and the responses were used as given. To assess fall experience, the participants were asked in each wave whether they had experienced a fall within the past 2 years, and the answers were used as given. Trouble with FOF was assessed by asking if FOF caused difficulties in their daily life activities, and the answers were used as given. Grip strength was analyzed using the average of measurements from the right and left hands and deemed to be weak at ≤24 kg for men and ≤ 15 kg for women (35). BMI was calculated using body weight and height (kg/m^2^). In this study, BMI was reclassified according to the WHO Asian Criteria of Obesity (36). The underweight (< 18.5 kg/m^2^), normal (18.5–22.9 kg/m^2^), and pre-obese (23.0–24.9 kg/m^2^) categories were grouped as non-obese (< 25 kg/m^2^), and the obese class I (25.0–29.9 kg/m^2^), obese class II (30.0–34.9 kg/m^2^), and obese class III (≥ 35 kg/m^2^) categories were grouped as obese (36, 37).

#### 2.3.3 Data analysis

The statistical analysis was conducted using IBM SPSS, version 28.0 (IBM Corp., Armonk, NY, USA). Descriptive statistics were performed for each variable using the frequency (%) for categorical variables, and mean and standard deviation (SD) for numerical variables. To determine the statistical significance of changes in ADL scores from wave 2 to wave 8, a repeated measure analysis of variance was performed. Changes in the frequency of ADL limitations during the eight waves are presented using Excel (Microsoft, Redmond, WA, United States). To identify factors associated with limitations in the total ADL score and the top three ADLs in wave 8, univariate and multivariate logistic analyses were performed. The independent variables reflected participant characteristics from wave 1, while the dependent variables are the ADL scores from wave 8. In the multivariate analysis, only variables that were significant in the univariate analyses were included using the forward method. Additionally, a sensitivity analysis was performed to investigate the effects of systematic selective attrition, which can occur due to missing follow-up during the data collection period, on ADL limitations. In this sensitivity analysis, 1,549 individuals were analyzed. This means that based on data from wave 8, we included 161 people who were excluded from the main analysis due to incomplete ADL data at waves 2, 3, 4, 5, 6, and 7 (Figure 1). The strength of the relationships was estimated as an odds ratio (OR) with 95% confidence interval (CI). Statistical significance was accepted at *p* < 0.05.

## 3 Results

**Figure 2:**
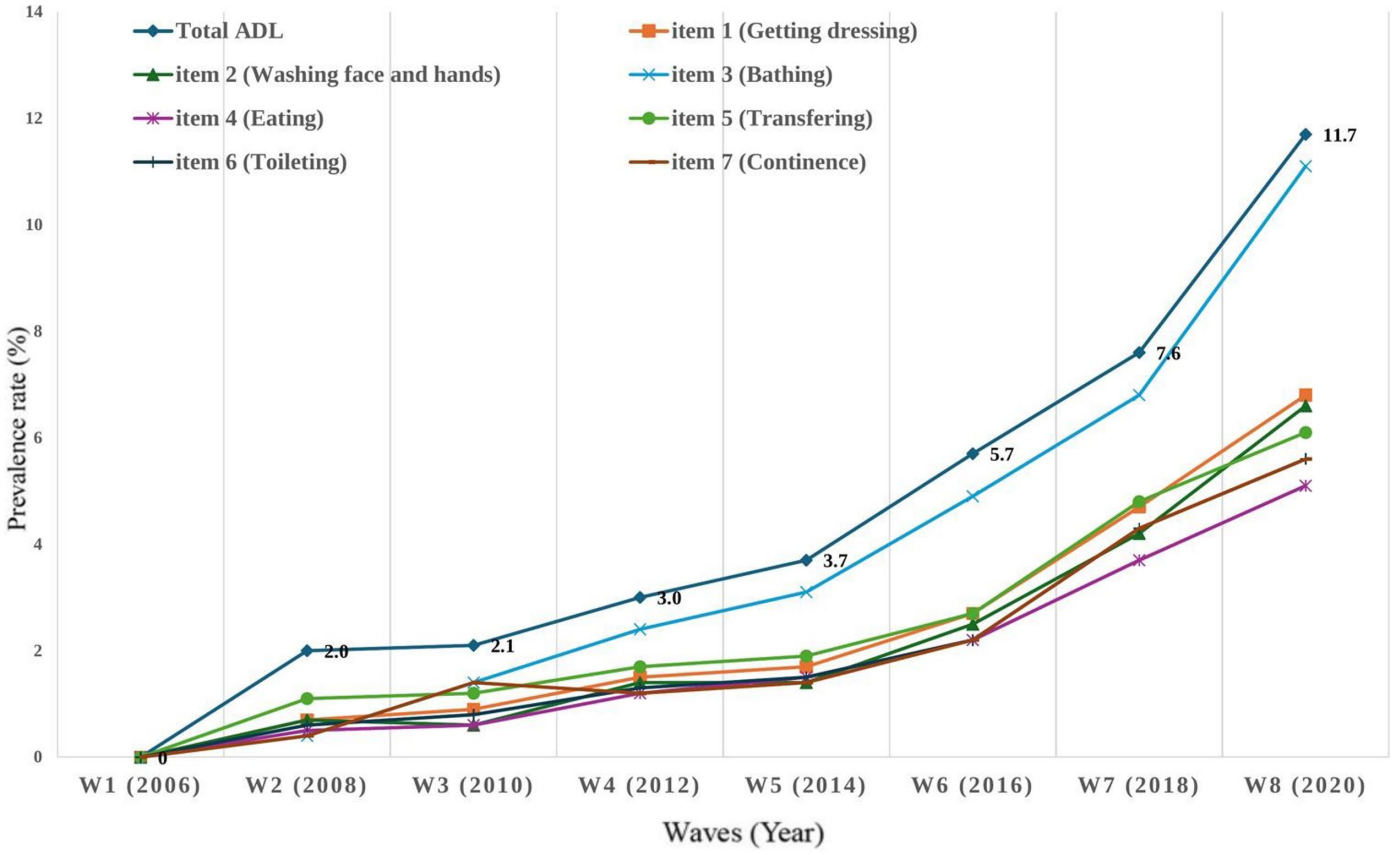
Changes in the prevalence of limitations in the activities daily living during 14 years.

The final analysis included 1,388 older adults and their baseline characteristics are shown in Table 1. In terms of demographic factors, the mean age was 69.88 years (SD = ±4.11), and 60.2% of them were female. 48.7% of the older adults were living with a spouse, 72.6% were married, 68.2% had 1–6 years of education, and 67.7% participated in social activity groups. In terms of health-related factors, more than half did not exercise regularly (63.8%), and the mean number of diagnosed chronic diseases was 0.98 (SD = ±0.98). Approximately one-third of the participants had dementia or mild cognitive impairment, 40.6% had poor hearing, and 78.7% had poor perceived vision. Perceived health was mostly poor (78.7%), with a mean perceived health was 60.87 (SD = ± 20.49), 15.9% had a fall experience within 2 years, and 23.1% had trouble with FOF. The average grip strength was 22.75 (SD = ±7.62), with 19.8% classified as having weak grip strength, and BMI was 21.4% obese. The overall trend in the prevalence of ADL limitations (total and each item) increased during the14 years of follow up, and the change in total ADL limitations was statistically significant (*F* = 54.40, *p* < 0.001) (Figure 2). The total prevalence of ADL limitations in wave 8 was 11.7%. The three ADLs with the highest frequency of limitation in wave 8 were bathing (ADL item 3, 11.1%), getting dressing (ADL item 1, 6.8%), and washing face and hands (ADL item 2, 6.6%), followed by transferring (ADL item 5, 6.1%), toileting (ADL item 6, 5.6%), continence (ADL item 7, 5.2%), and eating (ADL item 4, 5.1%).

**Table 1:** Characteristics of the participants at baseline (2006) (N = 1,388).

Factors	Variables	*n* (%)	M ± SD	Range
Demographic factors	Age (years)		69.88 ± 4.11	65–86
	65–75	1,184 (85.3)		
	>75	204 (14.7)		
	Sex			
	Female	836 (60.2)		
	Male	552 (39.8)		
	Living arrangement			
	Living with spouse and others	525 (37.8)		
	Living with spouse	676 (48.7)		
	Living alone	187 (13.5)		
	Marital status			
	Married	1,007 (72.6)		
	Separated/Divorced/Widowed or never married	381 (27.4)		
	Education (years)			
	More than 7 years	442 (31.8)		
	0–6	945 (68.2)		
	Participate in social groups			
	Yes	940 (67.7)		
	No	448 (32.3)		
Health related factors	Regular exercise			
	Yes	503 (36.2)		
	No	885 (63.8)		
	Number of chronic diseases		0.98 ± 0.98	0–6
	Cognition		24.62 ± 4.67	0–30
	Normal (≥ 24)	911 (65.6)		
	MCI (18-23)	331 (23.9)		
	Dementia (0–17)	132 (9.5)		
	Perceived hearing			
	Good	825 (59.4)		
	Poor	563 (40.6)		
	Perceived vision			
	Good	296 (21.3)		
	Poor	1,092 (78.7)		
	Perceived health			
	Good	336 (24.2)		
	Bad	1,052 (75.8)		
	Perceived QoL		60.87 ± 20.49	0–100
	Fall experience (within 2 years)			
	Yes	220 (15.9)		
	No	1,168 (84.1)		
	Trouble with FOF			
	Yes	321 (23.1)		
	No	1,067 (76.9)		
	Grip strength		22.75 ± 7.62	1.25–46.50
	Normal	1,021 (73.6)		
	Weakness	275 (19.8)		
	BMI		23.17 ± 2.75	13.84–36.33
	Non-obesity	1,035 (74.6)		
	Obesity	297 (21.4)		

Missing values: Education: (*n* = 1, 0.1%); Cognition: (*n* = 14, 1.0%); grip strength (*n* = 92, 6.6%); BMI (*n* = 56, 4.0%); MCI: mild cognitive impairment; QoL: quality of life; FOF: fear of falling; BMI: body mass index.

The univariate analyses of total ADL scores and the top three ADLs with limitation are presented in Table 2. Although the statistically significant predictors differed by total ADL and the top three items of ADL, the relatively common significant predictors were age 80–89 years, living with spouse, dementia, poor hearing, or weak grip strength.

**Table 2:** Univariate logistic regression model for total and top three ADLs with limitations (*N* = 1,388).

Variables^†^	Total ADL	Item 3 (Bathing)	Item 1 (Getting dressing)	Item 2 (Washing face and hands)
	OR (95% CI)	OR (95% CI)	OR (95% CI)	OR (95% CI)
Age (years)				
65–79 (ref)				
80–89	4.08 (2.84–5.88)^**^	4.19 (2.90–6.07)^**^	3.75 (2.39–5.88)^**^	3.17 (1.99–5.06)^**^
Sex				
Male (ref)				
Female	1.26 (0.90–1.77)	1.25 (0.88–1.78)	1.24 (0.80–1.92)	1.12 (0.72–1.73)
Living arrangement				
Living with spouse and others (ref)				
Living with spouse	0.53 (0.37–0.75)^**^	0.49 (0.34–0.71)^**^	0.53 (0.34–0.83)^**^	0.49 (0.31–0.77)^**^
Living alone	0.68 (0.41–1.14)	0.71 (0.43–1.19)	0.43 (0.20–0.91)^*^	0.43 (0.20–0.91)^*^
Marital status				
Married (ref)				
Separated/Divorced/Widowed or never married	1.74 (1.24–2.45)^**^	1.82 (1.28–2.57)^**^	1.69 (1.05–2.51)^*^	1.47 (0.94–2.31)
Education (years)				
More than 7 years (ref)				
0–6	1.68 (1.15–2.48)^**^	1.68 (1.13–2.49)^*^	1.17 (0.74–1.86)	1.18 (0.74–2.89)
Participate in social groups				
Yes (ref)				
No	1.30 (0.92–1.82)	1.27 (0.89–1.80)	1.53 (1.00–2.35)^*^	1.55 (1.01–2.39)^*^
Regular exercise				
Yes (ref)				
No	1.51 (1.06–2.17)^*^	1.53 (1.06–2.22)^*^	1.44 (0.91–2.29)	1.74 (1.07–2.82)^*^
Number of chronic diseases	1.19 (1.02–1.40)^*^	1.78 (1.01–1.38) ^**^	1.10 (0.90–1.36)	1.08 (0.88–1.33)
Cognition				
Normal (ref)				
MCI	1.37 (0.92–2.05)	1.43 (0.94–2.15)	1.31 (0.78–2.21)	1.12 (0.65–1.94)
Dementia	4.62 (2.99–7.12)^**^	4.44 (2.85–6.92)^**^	4.09 (2.40–6.95)^**^	4.30 (2.54–7.26) ^**^
Perceived hearing				
Good (ref)				
Poor	1.91 (1.37–2.65)^**^	1.95 (1.39–2.73)^**^	1.62 (1.09–2.51)^*^	1.54 (1.01–2.36)^*^
Perceived vision				
Good (ref)				
Poor	2.08 (1.28–3.38) ^**^	2.06 (1.25–3.39) ^**^	1.59 (0.89–2.85)	1.68 (0.92–3.06)
Perceived health				
Good (ref)				
Bad	1.88 (1.20–2.93)^**^	1.74 (1.11–2.72)^*^	1.38 (0.81–2.34)	1.23 (0.73–2.06)
Perceived QoL	0.99 (0.99–1.00)	1.00 (0.99–1.00)	1.00 (0.99–1.01)	1.00 (0.99–1.01)
Fall experience (within 2 years)				
No (ref)				
Yes	1.04 (0.51–2.14)	0.97 (0.46–2.06)	0.57 (0.18–1.84)	0.59 (0.18–1.91)
Trouble with FOF				
No (ref)				
Yes	1.90 (1.34–2.69)^**^	1.89 (1.32–2.71)^**^	1.53 (0.97–2.42)	1.70 (1.08–2.68)^*^
Grip strength				
Normal (ref)				
Weakness	2.16 (1.49–3.14)^**^	2.05 (1.40–3.01)^**^	1.95 (1.20–3.16)^**^	2.03 (1.25–2.30)^**^
BMI				
Non-obesity (ref)				
Obesity	1.29 (0.88–1.90)	1.27 (0.86–1.89)	1.26 (0.85–1.87)	1.23 (0.75–2.02)

OR, odds ratio; CI, confidence interval; ADL, activities of daily living; MCI, mild cognitive impairment; QoL, quality of life; FOF, fear of falling; BMI, body mass index; *p < 0.05, **p < 0.01. ^†^Data at baseline (2006).

The multivariate analyses of total ADL scores and the top three ADLs with limitation are presented in Table 3. The factors commonly associated with limitations in total ADL and the top three ADL limitations were age 80–89 years and dementia. Specifically, the factors associated with total ADL limitations were age 80–89 years (OR: 2.91, 95% CI: 1.93–4.40) or dementia (OR: 2.59, 95% CI: 1.55–4.31), poor vision (OR: 1.73, 95% CI: 1.02–2.92), or weak grip strength (OR: 1.52, 95% CI: 1.02–2.27). The factors associated with the top three ADLs were as follows: for bathing, age 80–89 years (OR: 3.01, 95% CI: 1.98–4.57), living with spouse (OR: 0.61, 95% CI: 0.41–0.92), dementia (OR: 2.77, 95% CI: 1.65–4.65), or poor vision (OR: 1.79, 95% CI: 1.04–3.07); for getting dressing, age 80–89 years (OR: 2.99, 95% CI: 1.80–4.98), or dementia (OR: 2.50, 95% CI: 1.34–4.66); for washing face and hands, age 80–89 years (OR: 2.35, 95% CI: 1.37–4.03), living alone (OR: 0.33, 95% CI: 0.13–0.81), or dementia (OR: 3.00, 95% CI: 1.61–5.59).

**Table 3:** Multivariate logistic regression model for total and top three ADLs with limitations (*N* = 1,388).

Variables^†^	Total ADL	Item 3 (Bathing)	Item 1 (Getting dressing)	Item 2 (Washing face and hands)
	OR (95% CI)	OR (95% CI)	OR (95% CI)	OR (95% CI)
Age (years)				
65–79 (ref)				
80–89	2.91 (1.93–4.40)^**^	3.01 (1.98–4.57)^**^	2.99 (1.80–4.98)^**^	2.35 (1.37–4.03)^**^
Living arrangement				
Living with spouse and others (ref)				
Living with spouse		0.61 (0.41–0.92)^*^		0.66 (0.41–1.08)
Living alone		0.66 (0.38–1.17)		0.33 (0.13–0.81)^*^
Cognition				
Normal (ref)				
MCI	1.16 (0.75–1.80)	1.25 (0.81–1.95)	1.19 (0.68–2.08)	0.91 (0.50–1.67)
Dementia	2.59 (1.55–4.31)^**^	2.77 (1.65–4.65)^**^	2.50 (1.34–4.66)^**^	3.00 (1.61–5.59)^**^
Perceived vision				
Good(ref)				
Poor	1.73 (1.02–2.92)^*^	1.79 (1.04–3.07)^*^		
Grip strength				
Normal (ref)				
Weakness	1.52 (1.02–2.27)^*^			

OR, odds ratio; CI, confidence interval; MCI, mild cognitive impairment; *p < 0.05, **p < 0.01. ^†^ Data at baseline (2006).

The results of the sensitivity analysis were similar to the main results. In the univariate analyses, the variables that commonly limited total ADLs, as well as bathing, getting dressing, and washing face and hands were age 80–89 years, separated/divorced/widowed or never married, no regular exercise, dementia, poor hearing, poor vision, trouble with FOF, and weak grip strength. Age 80–89 years, dementia, poor hearing, or weak grip strength were common factors associated with ADL limitations in both the main and sensitivity analyses, while several additional variables were significant in the sensitivity analysis (Supplementary Table S1). The multivariate analysis results were also similar to the main results, as shown in Supplementary Table S2. Total ADLs were most commonly limited by age 80–89 years (OR: 3.11, 95% CI: 2.12–4.55), dementia (OR: 2.26, 95% CI: 1.39–3.69), poor vision (OR: 1.71, 95% CI: 1.04–2.82), or weak grip strength (OR: 1.73, 95% CI: 1.18–2.53). Bathing was most commonly limited by age 80–89 years (OR: 3.10, 95% CI: 2.10–4.59), dementia (OR: 2.19, 95% CI: 1.32–3.62), poor vision (OR: 1.78, 95% CI: 1.06–2.99), or weak grip strength (OR: 1.58, 95% CI: 1.06–2.35). Getting dressing was most affected by age 80–89 years (OR: 3.54, 95% CI: 2.25–5.57), not participating in social groups (OR: 1.59, 95% CI: 1.04–2.44), or weak grip strength (OR: 2.00; 95% CI: 1.26–3.15). Washing face and hands was typically affected by age 80–89 years (OR: 2.55, 95% CI: 1.57–4.13), dementia (OR: 2.24, 95% CI: 1.24–4.04), or weak grip strength (OR: 1.93, 95% CI: 1.20–3.10). Age 80–89 years was a common factor in both the main and sensitivity analyses, while weak grip strength was an additional common factor in the sensitivity analysis.

## 4 Discussion

This study used data from a representative longitudinal cohort study in Korea to examine trends in total and individual ADL scores, identify the ADLs most reported as challenging, and analyze factors that predict functional limitations among community-dwelling older adults. To the best of our knowledge, this study is the first to longitudinally investigate changes and influencing factors for individual ADLs in Korea. Therefore, the results of this study can be used to improve the QoL for older adults with functional limitations and reduce the burden on the health and welfare system tasked with managing and customizing care for that population by elucidating the complex factors that individual ADL limitations over time.

### 4.1 Trends of ADL limitation

The results of this study indicate that the prevalence of total ADL limitations has consistently increased over time, particularly since 2014 (wave 5). This can be interpreted as a sharp increase in ADL limitations among individuals aged 73 or older (or in their mid-70s), which corresponds to adding 8 years to the baseline age of 65 in 2006. It is well-established that functional limitations increase with age, and the results of this study are consistent with the previous studies (8, 38). The significant increase in the prevalence of ADL limitations in the later stages of this study aligns with the 3-year and 6-year follow-up results from the Swedish National Study on Aging and Care-Kungsholmen, which analyzed individuals aged 60 years and older (38). According to Santoni et al. (38), fewer than 10% of participants younger than 85 years had functional limitations, but comprehensive health functional decline, including ADL limitations, accelerated rapidly after age 85 years. The concordance between those previous studies and our results underscores the importance of timely and targeted interventions to manage the health and functional status of the oldest old people, as opposed to younger old people. This study contributes to the body of evidence that highlights the need to develop individualized ADL limitation management and treatment plans that address the heterogeneity of the disability process and the complex health needs of older adults to manage and prevent disability in that population.

Specifically, comparing the prevalence of ADL limitations in community-dwelling people aged 65 and older from this study with those from other nationally representative aging studies, our results (11.7% in wave 8) are similar to the 13% reported in the Irish Longitudinal Study on Aging (TILDA) (20). However, the TILDA results (20) are based on a cross-sectional inferential study using six ADL items, so the study design and methodology have some differences from those used in this research. Therefore, comparisons between the TILDA results and those from this study should be made with caution.

The results of previous nationally representative longitudinal cohort studies of ADL limitation prevalence are as follows: Population Study of Chinese Elderly in Chicago (PINE), from 7.1% at baseline to 8.3% after 2 years (*n* = 282); In a study analyzing the China Health and Retirement Longitudinal Study (CHARLS) and Survey of Health, Ageing and Retirement in Europe (SHARE), the baseline prevalence of ADL limitations was reported as 9.77% in CHARLS and 5.05% in SHARE (39). After 4-year follow-up, the result showed an increased prevalence of ADL limitations, particularly among older adults with multimorbidity (older adults without chronic diseases vs. with 4 or more chronic diseases, CHARLS 8.38% vs. 35.52%, SHARE 3.87% vs. 19.49%) (39). However, all those studies analyzed participants with and without functional limitations at baseline, which is different from this study, which excluded participants with functional limitations at baseline. In addition, the age criteria for participants in the PINE, CHARLS, and SHARE studies were 60 years and older (mean 78.6), 45 years and older (mean 56.0), and 50 years and older (mean 62), respectively, and their longitudinal observation periods were 2, 4, and 4 years, respectively. Those criteria also differ from this study [65 years and older (mean 69.88), 14 years of observation], making it difficult to completely compare the prevalence of functional limitations with those reported in previous studies.

The longitudinal prevalence of ADL limitations in Japan (40), which is geographically close to Korea, was 38.4% after 9 years, which is higher than the 11.7% observed in this study after 14 years. The Japanese study, like ours, excluded participants with ADL limitations at baseline, but it was a prospective, population-based cohort study of osteoporosis and osteoarthritis in women aged 40 and older. Therefore, the presence of diseases that make women particularly vulnerable to disability might have influenced the increase in functional limitations. Furthermore, the observation period (9 years) was shorter than in our study, and the sample size was only 264 participants. Given those differences, comparisons with our study results should be made cautiously.

Various previous studies have evaluated functional limitations, but variability in the assessment methods, clinical settings, and population diversity make it difficult to interpret and compare results (41). The different contextual definitions of functional limitation and variations in measurement tools based on different disability models (42, 43) further complicate the comparison and interpretation of study results, presenting significant challenges for researchers, policymakers, and practitioners alike (43). Therefore, to more accurately understand and assess functional limitations across different contexts and cultures, future research should use integrative approaches to overcome the diversity and complexity of measuring functional limitations and generate internationally comparable data (43, 44).

In our wave 8 data, the order of highest prevalence for limitations in individual ADLs was bathing, getting dressed, washing face and hands, transferring, toileting, continence, and eating. Bathing consistently had the highest ADL limitation prevalence from wave 2 to wave 8, with more than 1 in 10 older adults (11.1%) experiencing functional limitation in bathing in wave 8. These results suggest that special attention and support are needed for bathing. It is especially important to recognize that the rate of functional limitation accelerates with age.

In the literature, bathing is also consistently reported to have the most challenging functional limitations (45–47). A study (47) based on data from the Health and Retirement Study (HRS) in the United States (2006–2014), KLoSA (2006–2014), and the Japanese Study of Aging and Retirement (2007–2013) found that bathing and dressing consistently had the highest prevalence of functional limitations among the ADLs in all three countries. On the other hand, eating is the ADL that is maintained for the longest time and can thus be considered to cause the least disability (48, 49). These findings suggest that the Korean older adults analyzed in this study experience ADL limitations that are consistent with international trends. However, unlike the results of this study, the highest prevalence of ADL limitations was reported differently in the Netherlands (taking care of feet and toenails) (8), Ireland (dressing) (20), China (stair climbing) (50), and a systematic literature review (personal hygiene, walking, and transferring) (51). These conflicting findings could be due to differences in the physical, social, and environmental conditions of the populations and the research methods used. The context and conditions of each study must be carefully considered when analyzing differences among findings because they have important implications for developing strategies to prevent and manage functional limitations.

The fact that bathing is a commonly reported ADL with a high prevalence of limitation in both this study and several previous studies can be explained by the WHO’s International Classification of Functioning, Disability and Health disability framework, which emphasizes the role of environmental factors as significant contributors to disability (1, 44, 52). Difficulty with bathing arises from a complex interplay of personal factors (physical functional limitations) and environmental barriers (physical environment, social support, and lack of institutional services) (1, 44, 52). Those factors collectively make it challenging for older adults to engage in daily hygiene activities such as bathing and thereby exacerbate functional limitations and contribute to the onset of disability (1, 44). Additionally, the HRS panel data analysis indicates that among ADLs, functional limitation in bathing is the strongest predictor of the risk of institutionalization (53). However, ADL limitations in older adults are dynamic and potentially reversible. Previous studies have shown that the onset of functional limitation can be reversed or diminished over time as part of the aging process (54, 55). Furthermore, a study investigating the trajectories of ADLs in older adults reported that although most trajectory transitions indicated worsening, 45% showed recovery (54). Therefore, accurately assessing the condition of older adults with ADL limitations and providing appropriate healthcare and environmental support could recover ADL limitations.

According to a representative study, adults in the U.S. population aged 65 and older do not get to live independently and safely due to a lack of supportive assistive devices (56). In response, the WHO is supporting the Global Cooperation on Assistive Technology (GATE) initiative to ensure that people worldwide who need assistive technology have access to appropriate devices and services (11). Identifying environmental barriers such as the absence of grab bars in bathrooms, shower seats, and non-slip mats and improving social support through the development of bath-related assistive technology is essential for preventing functional limitations and disabilities.

Accordingly, assistive technology has been increasingly emphasized as an important means of reducing the burden of daily activities for older adults with functional limitations and maintaining or enhancing their independence (1, 56, 57). Korea is also promoting the development and commercialization of assistive technology to support people with functional limitations in various daily activities, but there is a lack of access to such services and a lack of diversity in assistive devices (58). Barriers to the sustainable use of assistive technology include the capacity of service providers and aftercare systems (58, 59). The WHO’s GATE 5P framework emphasizes five key elements, People, Policy, Products, Service Provision, and Personnel, as a strategic framework for enhancing access to assistive technology (60). Therefore, in planning strategies to advance assistive technology for people with disabilities, it is necessary to understand the dynamics of their daily lives challenges and use an integrated approach to build systems to address those challenges.

### 4.2 Predictive factors

In this study, the strongest predictor of total ADL disability was old age, followed by dementia, poor vision, and low grip strength. The predictors common to the three ADLs with the highest limitation were old age and dementia. A major new finding from this study is that age and cognition were significant predictors of both total ADL limitation and the three individual ADLs with the highest prevalence of limitation.

Age and cognition are already well known as important conditions associated with total ADL limitation in community-dwelling (17), hospitalized (50), and institutionalized older adults (61). Increasing age is accompanied by physical and cognitive decline (62), and dementia is a major geriatric disease whose incidence increases with age, causing not only cognitive decline but also physical decline. In other words, memory decline can impair the ability to remember, plan, and carry out daily activities (17). Our findings are consistent with those of previous studies and support the notion that age and cognitive decline are important factors that significantly affect total ADL scores.

Age and cognition were consistently significant predictors of total ADL limitations and the three ADLs with the highest prevalence of limitation in this study, but previous studies (20, 63) that examined predictors of Instrumental Activities of Daily Living (IADL) limitation reported different results. For example, a recent study using KLoSA data analyzed the predictors of IADL limitation in community-dwelling older adults and found that the predictors for limitations in total IALD and the top three ranked items of IADLs varied (63). In addition, an Irish longitudinal study on ageing (20) found that cognition was not significant for ADL limitation but was significant for IADL limitation.

The different results between this study and previous studies (20, 63) need to be interpreted from several important perspectives. First, they could be due to diversity in the functional scope and skills required in the ADL and IADL assessments (48, 64). ADLs are activities necessary for managing basic physical needs; they are closely related to an individual’s fundamental physiological needs and are essential for maintaining daily independence (48). In contrast, IADLs include more complex activities that enable an individual to live independently in the community, such as using transportation, managing medications, and shopping, which require a range of social and cognitive skills (64).

Second, it is generally recognized that ADL limitations reflect physical health problems, whereas IADL limitations are sensitive to cognitive decline. However, given the progression of IADL limitations to ADL limitations, it is important to consider that cognitive function also plays an important role in ADL limitations (48). In other words, IADL limitations precede ADL limitations, and so cognitive function affects IADLs in an early stage of decline, but as functional limitations progress, declines in cognitive function such as reasoning and planning also significantly affect ADLs (48). The findings of this study highlight the need for policymakers and health professionals to consider both the physical and cognitive components of aging when assessing ADL limitations in older adults and building support systems. In particular, they confirm the importance of early intervention programs to minimize the impact of cognitive decline on ADLs in older adults.

Another major new finding of this study is that grip strength predicted only total ADL limitation; it was not associated with the three ADLs with the highest prevalence of limitation. Because it represents overall muscle strength, grip strength is an important indicator used to identify old adults at high risk of physical disability (65). However, in the final model in this study, grip strength was not a significant predictor of total ADL limitations or the three ADLs most limited, possibly due to its general representativeness of overall physical function rather than specific activities. Activities such as bathing, dressing, and washing face and hands might rely more on coordination and cognitive judgment than on muscle strength. The influence of muscle strength might vary by body part, and grip strength might not significantly affect activities in which the lower body plays a crucial role. Consistent with our findings, previous studies have identified grip strength as a factor for total ADL limitations (40, 46, 65). Although few studies have explored the relationship between grip strength and individual ADLs, an older study (66) examined this relationship and found that low grip strength predicted functional limitations in dressing and bathing 25 years later. Therefore, it is essential to focus on the prevention of grip strength decline in older adults, further longitudinal studies are needed to obtain stable results about the relationship between grip strength and individual ADLs. Those results could further clarify the potential of grip strength to predict individual ADL limitations and contribute to the development of intervention strategies.

This study found that poor vision significantly contributes to total ADL and bathing limitations. This result is consistent with previous research (16), confirming that poor vision increases ADL limitations by making safe mobility difficult. Therefore, attention should be given to preventing vision decline in older adults as a modifiable predictor. Additionally, our results showed that living with a spouse had a positive effect on bathing by reducing the likelihood of ADL limitations, which may be related to the physical or emotional support provided by the spouse. On the other hand, living alone reduced the likelihood of ADL limitations in washing face and hands, which may reflect the tendency of older adults living alone to maintain or enhance function because they must perform all activities on their own (16). Therefore, further exploration of living arrangements and ADL limitations will be necessary.

Although they were not identified as final influencing factors for ADL limitations in this study, factors such as female (16, 32), separated/divorced/widowed and never married (16), multimorbidity (17, 39), poor perceived health (18, 67), and pain (20, 40, 45) have consistently been reported as influencing ADL limitations among community-dwelling older adults. The differences between those results and ours might be influenced by external variables such as the characteristics of the study participants, the distribution of underlying diseases, socioeconomic factors, regional characteristics, differences in the variables controlled in the study design, and differences in measurement tools. The lack of a significant association between sex and ADL limitations in this study may be due to the pronounced influence of age and cognitive function over the long-term follow-up period, which could have relatively diminished the effect of sex. Additionally, the lack of significance of marital status in relation to ADL limitations may suggest that support from family, relatives, and social networks has a more substantial impact on ADL than marital status alone. In Korea, family support for older adults tends to be strong regardless of marital status, and there is a national focus on developing family and social support systems for older adult care (27). As such, differences in ADL limitations based on marital status may not have been prominent in the present study as cases without a current married status (separated/divorced/widowed or never married) were combined for analysis. This combination limits the ability to clearly identify specific differences in the impact on ADL limitations between the separated/divorced/widowed and never married groups. Therefore, future longitudinal studies are needed to investigate the causal relationships among the various factors that influence ADL disabilities in older adults from diverse cultural backgrounds. Such approaches and studies will significantly contribute to the understanding of health in older adults and the development of effective strategies for preventing and intervening in ADL limitations.

### 4.3 Limitation

This study has some limitations. First, the KLoSA data are based on self-report, which introduces the limitation of subjective assessment. It is important to consider that perceptions of “difficulty” with a particular task can vary among individuals and that the predictors of objective and subjective assessments of disability can differ (66). Second, sample attrition during the follow-up period, which tends to be a problem in studies of older adults, is another limitation of this study. We were somewhat reassured when the result of the sensitivity analyses, we conducted to assess the impact of missing data on the findings were similar to the main results. Therefore, while the impact of missing participants on the main analysis results appears to be minimal, the significant differences in certain variables (e.g., participation in social group) on specific items in the sensitivity analysis suggests that the subtle effects of participant attrition on study outcomes cannot be ruled out. Third, previous studies have shown that the progression of functional limitations in the older adults differs depending on the severity, with a slower progression in the early years and a more rapid progression in later years (68). However, we did not consider differences in the time course according to the severity of functional limitations. Therefore, we suggest that future studies consider temporal trends according to the severity of functional limitations in community-dwelling older adults. Finally, since this study excluded individuals with ADL limitations at baseline, the trend estimates may be overestimated as they reflect only healthy adults without functional limitations. This is because the statistical predictions only consider changes in the direction of worsening limitations. Therefore, it is necessary to interpret the results conservatively.

## 5 Conclusion

This study has identified the trends and predictors of changes in total ADL limitations and the three ADLs with the highest prevalence of limitation among community-dwelling older adults in Korea. The results of this study provide insights for healthcare professionals and policymakers, showing the importance of assessing not only the total ADL score but also the difficulties experienced with each ADL when planning and providing rehabilitation services for older adults with ADL limitations. Additionally, the findings demonstrate how ADL limitations change with increasing age, emphasizing the need for rehabilitation and healthcare services focused on the ADL items with the highest prevalence of limitation, especially among the oldest adults. Finally, this study highlights that bathing is the most challenging ADL for older adults, suggesting that as age increases, physical and social supports, policies, and services need to be considered comprehensively, including the application of integrated assistive technologies.

## Data Availability

Publicly available datasets were analyzed in this study. This data can be found here: https://survey.keis.or.kr/eng/klosa/klosa01.jsp.
