# Supplementary material for: Longitudinal trends and predictors of limitations in activities of daily living in community-dwelling older adults: evidence from the KLoSA study
Source: Front Public Health. 2024 Dec 13;12:1485732. doi: 10.3389/fpubh.2024.1485732 (PMC11673221; doi:10.3389/fpubh.2024.1485732)
Supplement: Supplementary file 1 [file Supplementary_file_1.docx]

Table S1. Univariate logistic regression model for total and top three ranked ADL limitations including older adults who did not participate during waves 2–7 (N= 1,549)

| Variables^†^ | Total ADL | Item 3  (Bathing) | Item 1  (Getting dressing) | Item 2  (Washing face and hands) |
| --- | --- | --- | --- | --- |
|  | OR (95% CI) | OR (95% CI) | OR (95% CI) | OR (95% CI) |
| Age (years) |  |  |  |  |
| 65-79 (ref) |  |  |  |  |
| 80-89 | 4.42 (3.16-6.20)^**^ | 4.45 (3.15-6.29)^**^ | 4.33 (2.88-6.50)^**^ | 3.69 (2.43(5.61)^**^ |
| Sex |  |  |  |  |
| Male (ref) |  |  |  |  |
| Female | 1.22 (0.89-1.68) | 1.24 (0.89-1.72) | 1.22 (0.81-1.82) | 1.14 (0.76-1.72) |
| Living arrangement | |  |  |  |
| Living with spouse and others |  |  |  |  |
| Living with spouse | 0.53(0.38-0.75)^**^ | 0.52(0.36-0.73) ^**^ | 0.55(0.36-0.83)^**^ | 0.52 (0.34-0.79)^**^ |
| Living alone | 0.82(0.52-1.30) | 0.80 (0.50-1.29) | 0.57(0.30-1.09) | 0.58 (0.31-1.11) |
| Marital status |  |  |  |  |
| Married (ref) | |  |  |  |
| Separated/Divorced/  Widowed or never married | 1.89 (1.38-2.59)^**^ | 1.94 (1.40-2.69)^**^ | 1.81 (1.22-2.69)^**^ | 1.70 (1.14-2.55)^*^ |
| Education (years) |  |  |  |  |
| More than 7 years (ref) |  |  |  |  |
| 0-6 | 1.70 (1.19-2.43)^**^ | 1.75 (1.20-2.54)^**^ | 1.34 (0.87-2.07) | 1.34 (0.86-2.07) |
| Participate in social groups | |  |  |  |
| Yes (ref) |  |  |  |  |
| No | 1.32 (0.96-1.82) | 1.28 (0.92-1.78) | 1.67 (1.13-2.47)^*^ | 1.65 (1.11-2.46)^*^ |
| Regular exercise |  |  |  |  |
| Yes (ref) |  |  |  |  |
| No | 1.56 (1.11-2.20)^*^ | 1.63 (1.14-2.33)^**^ | 1.58 (1.03-2.44)^*^ | 1.94 (1.23-3.08)^**^ |
| Number of chronic diseases | 1.20 (1.04-1.39)^**^ | 1.18 (1.01-1.37)^*^ | 1.14 (0.95-1.37) | 1.11 (0.92-1.35) |
| Cognition |  |  |  |  |
| Normal (ref) |  |  |  |  |
| MCI | 1.53 (1.05-2.21)^**^ | 1.52 (1.04-2.24)^*^ | 1.54 (0.96-2.46) | 1.31 (0.80-2.14) |
| Dementia | 4.54 (3.02-3.84)^**^ | 4.31 (2.83-6.56)^**^ | 4.35 (2.66-7.10)^**^ | 4.53 (2.79-7.37)^**^ |
| Perceived hearing | |  |  |  |
| Good (ref) |  |  |  |  |
| Poor | 1.97 (1.45-2.68)^**^ | 1.97 (1.43-2.70)^**^ | 1.83 (1.24-2.69)^**^ | 1.69 (1.14-2.51) ^**^ |
| Perceived vision | |  |  |  |
| Good (ref) |  |  |  |  |
| Poor | 2.20 (1.38-3.49)^**^ | 2.24 (1.38-3.64)^**^ | 1.99 (1.12-3.53)^*^ | 2.08 (1.15-3.76)^*^ |
| Perceived health |  |  |  |  |
| Good (ref) |  |  |  |  |
| Bad | 2.02 (1.33-3.07)^*^ | 1.83 (1.20-2.79)^**^ | 1.48 (0.91-2.42) | 1.33 (0.82-2.15) |
| Perceived QoL | 0.99 (0.99-1.00) | 1.00 (0.99-1.00) | 1.00 (0.99-1.01) | 1.00 (0.99-1.01) |
| Fall experience (within 2 years) | |  |  |  |
| No (ref) |  |  |  |  |
| Yes | 1.18 (0.63-2.22) | 1.16 (0.61-2.23) | 0.95 (0.40-2.22) | 0.99 (0.42-2.32) |
| Trouble with FOF |  |  |  |  |
| No (ref) |  |  |  |  |
| Yes | 1.86 (1.34-2.58)^**^ | 1.82 (1.30-2.55) ^**^ | 1.53 (1.01-2.33)^*^ | 1.70 (1.12-2.58)^*^ |
| Grip strength |  |  |  |  |
| Normal (ref) |  |  |  |  |
| Weakness | 2.49 (1.75-3.55)^**^ | 2.30 (1.60-3.33)^**^ | 2.53 (1.62-3.95)^**^ | 2.52 (1.62-3.92)^**^ |
| BMI |  |  |  |  |
| Non-obesity (ref) |  |  |  |  |
| Obesity | 1.22 (0.85-1.75) | 1.23 (0.85-1.79) | 1.35 (0.87-2.09) | 1.24 (0.78-1.95) |

OR: odds ratio; CI: confidence interval; ADL: activities of daily living; MCI: mild cognitive impairment; QoL: quality of life; FOF: fear of falling; BMI: body mass index; **p* < .05, ***p* < .01. ^†^ Data at baseline (2006).

Table S2. Multivariate logistic regression model for in ADL limitations including older adults who did not participate during waves 2–7 (N= 1,549)

| Variables^†^ | Total ADL | Item 3  (Bathing) | Item 1  (Getting dressing) | Item 2  (Washing face and hands) |  |
| --- | --- | --- | --- | --- | --- |
|  | OR (95% CI) | OR (95% CI) | OR (95% CI) | OR (95% CI) |  |
| Age (years) |  |  |  |  |  |
| 65-79 (ref) |  |  |  |  |  |
| 80-89 | 3.11 (2.12-4.55)^**^ | 3.10 (2.10-4.59)^**^ | 3.54 (2.25-5.57)^**^ | 2.55 (1.57-4.13)^**^ |  |
| Participate in social groups |  |  |  |  |  |
| Yes (ref) |  |  |  |  |  |
| No |  |  | 1.59 (1.04-2.44) ^*^ |  |  |
| Cognition |  |  |  |  |  |
| Normal (ref) |  |  |  |  |  |
| MCI | 1.26 (0.84-1.88) | 1.26 (0.83-1.92) |  | 0.97 (0.56-1.67) |  |
| Dementia | 2.26 (1.39-3.69)^**^ | 2.19 (1.32-3.62) ^**^ |  | 2.24 (1.24-4.04)^**^ |  |
| Perceived vision |  |  |  |  |  |
| Good (ref) |  |  |  |  |  |
| Poor | 1.71 (1.04-2.82)^*^ | 1.78 (1.06-2.99)^*^ |  |  |  |
| Grip strength |  |  |  |  |  |
| Normal (ref) |  |  |  |  | |
| Weakness | 1.73 (1.18-2.53)^**^ | 1.58 (1.06-2.35) ^*^ | 2.00 (1.26-3.15) ^**^ | 1.93 (1.20-3.10) ^**^ | |

OR: odds ratio; CI: confidence interval; MCI: mild cognitive impairment; **p* < .05, ***p* < .01. ^†^ Data at baseline (2006).
